# Skiagraphy of the Urinary Bladder

**Published:** 1905-12

**Authors:** 


### Skiagraphy of the Urinary Bladder.

Drs. Voelcker and Lichtenberg (*Münch. Med. Wochenschrift,* August 15, 1905.) publish from Czerny's Surgical Clinic at Heidelberg University, a paper on the shape of the urinary bladder as revealed by skiagraphy. The ordinary means of examination as well as cystoscopy give but little information in regard to the shape of the organ. The soluble bismuth compounds are too poisonous to inject into the bladder for the purpose of rendering it opaque, and the insoluble ones are liable to leave material that may form the nucleus of calculi. 2% collargolum solutions are, however, eminently adapted for the purpose, for they do not irritate the vesical mucosa. On the contrary, in some cases of chronic cystitis there was marked improvement after collargolum injection. They have made extensive use of this accidental discovery, as the injection causes no pain whatever, and employ collargolum in cystitis exclusively in the place of other antiseptics, such as silver nitrate, which is notoriously painful. In chronic cystitis from prostatic hypertrophy, for instance, they inject 3½ ozs. of a 1% collargolum solution, which may be left in the bladder as long as desired, and even permanently.

Excellent skiagraphs were obtained, giving valuable information in prostatic hypertrophy, vesical prolapse in the female, and other displacements and deformities of the organ. The article is illustrated.

